# Expression of serum *miR*‐*135a* in patients with allergic rhinitis and its relationship with Treg/Th17 balance

**DOI:** 10.1002/kjm2.12918

**Published:** 2024-12-11

**Authors:** Jing Cai, Fang Wang, Sheng‐Liu Shi

**Affiliations:** ^1^ Department of Octolaryngology First Affiliated Hospital of Kunming Medical University Kunming People's Republic of China; ^2^ Department of Rehabilitation Medicine First Affiliated Hospital of Kunming Medical University Kunming People's Republic of China

**Keywords:** allergic rhinitis, cell balance, microRNA, microRNA‐135a, regulatory T cell/T helper cell 17

## Abstract

The dysregulation of microRNA (miRNA) expression contributes to the development of allergic rhinitis (AR). This study investigates serum *miR‐135a* levels and their association with regulatory T cell (Treg) and T helper cell 17 (Th17) balance in AR patients. A total of 93 AR patients and 76 healthy controls were retrospectively recruited. Levels of serum *miR‐135a*, peripheral blood Th17 and Treg cells, and Treg/Th17‐related cytokines were measured. We assessed the diagnostic value of serum *miR‐135a* for AR and its relationship with Treg/Th17 balance. AR patients showed significantly elevated immunoglobulin E (IgE), peripheral blood Th17 cells, and IL‐17 and IL‐6 levels, alongside reduced serum *miR‐135a*, Treg cells, IL‐10, TGF‐β1, and Treg/Th17 ratios. A serum *miR‐135a* of ≤0.536 demonstrated diagnostic potential for AR. Patients with higher serum *miR‐135a* levels displayed increased Treg cell level and Treg/Th17 ratios, reduced Th17 cell, and lower total nasal symptom score (TNSS). Serum *miR‐135a* levels in AR patients negatively correlated with TNSS, IL‐17, IL‐6, and Th17 cell percentages, and positively correlated with IL‐10, TGF‐β1, Treg cell percentages, and Treg/Th17 ratios. Collectively, decreased serum *miR‐135a* levels in AR patients are associated with Treg/Th17 balance, supporting *miR‐135a* as a potential biomarker for AR diagnosis.

## 1 INTRODUCTION

Allergic rhinitis (AR) is a widespread allergic disorder affecting approximately 10%–40% of the global population.^1^ It is characterized by symptoms such as sneezing, nasal itching, congestion, and rhinorrhea. AR is associated with various complications and serves as a significant risk factor for poor asthma management by impacting both the upper and lower respiratory tracts.^2^ Current clinical treatments for AR, including antihistamines and intranasal steroids, mainly target symptomatic relief; however, specific immunotherapy has limitations that restrict its broad application.^3^ The pathogenesis of AR involves a complex interplay of genetic and environmental factors,^4^ though the underlying mechanisms remain incompletely understood. To better understand AR and improve treatment options, identifying biomarkers associated with its onset and progression is critical, as is exploring novel therapeutic targets that offer greater specificity.

AR is primarily mediated by immunoglobulin E (IgE), with the immune response acting as the key pathological driver.^5^ Regulatory T cells (Treg) are essential for maintaining immune tolerance to allergens, and their function is often compromised in AR patients.^6^ Treg cells secrete interleukin (IL)‐10, a cytokine significantly reduced in AR, possibly due to Treg dysfunction, which contributes to immunodeficiency in these individuals.^7^ Transforming growth factor β1 (TGF‐β1) is vital for Treg and T helper 17 (Th17) cell differentiation.^8^ Th17 cells, characterized by the production of IL‐17 and IL‐6, contribute to inflammatory responses in AR. Increased IL‐17 levels stimulate Th2 cells, resulting in elevated IgE levels and recruitment of eosinophils and neutrophils.^9^ These cytokines stimulate IgE production by B cells, which is central to AR pathogenesis.^10^

MicroRNAs (miRNAs) are a class of non‐coding, single‐stranded RNAs that play a pivotal role in regulating gene expression. They function by binding to target mRNAs, leading to either mRNA degradation or the inhibition of protein translation.^11^ miRNAs are involved in modulating allergic inflammation through their influence on immune cell activity.^12^ Notably, *miR‐135a* expression is reduced in an AR mouse model, suggesting a role in AR pathogenesis.^13^ *miR‐135a* suggesting a role in AR pathogenesis T‐cell differentiation and plasticity by targeting GATA3, a critical transcription factor in immune cell function.^14^ Additionally, previous studies indicate that *miR‐135a* helps to correct the Th1/Th2 imbalance in AR mice, contributing to immune regulation in allergic conditions.^15^

However, limited research has examined serum *miR‐135a* expression in AR patients and its potential impact on Treg/Th17 balance. Given the involvement of Treg and Th17 cells in immune regulation, understanding the relationship between miR‐135a and Treg/Th17 balance may provide valuable insights into AR mechanisms. Thus, this study aims to investigate the expression of serum *miR‐135a* in AR patients and explore its association with Treg/Th17 cell balance, potentially identifying miR‐135a as a biomarker and therapeutic target in AR.

## 2 MATERIALS AND METHODS

### 2.1 Ethics statement

This study received approval from the Ethics Committee of our hospital and adhered to the principles outlined in the Declaration of Helsinki.

### 2.2 Study subjects

We retrospectively selected a cohort of 124 AR patients treated at the Otolaryngology Clinic of our hospital between February 2020 and September 2023. After applying inclusion and exclusion criteria, 93 AR patients were included as the AR group. Additionally, 76 healthy individuals, with no family history of allergies, were recruited as the control group. There were no significant differences between the groups in terms of sex, age, body mass index (BMI), or smoking history.

### 2.3 Inclusion and exclusion criteria

The inclusion criteria comprised of the following^1^: diagnosis based on the allergic rhinitis and its impact on asthma (ARIA) guidelines,^16^ confirmed by symptoms, physical examination, skin prick test, and specific IgE test^2^; presence of symptoms such as nasal itching, nasal congestion, paroxysmal sneezing, and rhinorrhoea to varying degrees^3^; age 18 years or older^4^; and availability of complete clinical data.

The exclusion criteria were as follows^1^: history of other allergic or respiratory conditions or use of medications within the last 4 weeks^2^; conditions including vasomotor rhinitis, non‐AR with eosinophilia syndrome, infectious rhinitis, hormonal rhinitis, drug‐induced rhinitis, or other nasal disorders^3^; recent history of infectious disease or the use of immunosuppressant medication^4^; presence of malignant tumors.

### 2.4 Data collection

Collected data included patients' age, sex, BMI, disease course, specific IgE level, total nasal symptom score (TNSS), and symptom onset duration [categorized as intermittent AR (symptoms <4 days/week or duration <4 weeks) or persistent AR (symptoms ≥4 days/week or duration ≥4 weeks)]. Fasting elbow venous blood samples (3 mL) were obtained from all subjects and processed for necessary testing.

### 2.5 Detection of peripheral blood Th17 and Treg cells

Th17 and Treg cell percentages were measured using a flow cytometer (cytoflex, Beckman Coulter, Chaska, MN, USA) and analyzed with Kaluza 1.2 software (Beckman Coulter). Antibodies and reagents were obtained from Biolegend (San Diego, CA, USA).

Peripheral blood Th17 was assayed. In brief, fasting venous blood (2 mL) was collected in the morning, and peripheral blood mononuclear cells (PBMCs) were isolated using human lymphocyte isolation solution. PBMC concentration was adjusted to 1 × 10^7^/mL with RPMI1640 medium, and 100 μL of PBMC suspension was cultured. The culture was supplemented with phorbol 12‐myristate 13‐acetate, ionomycin and monensin, then incubated at 37°C in 5% CO_2_ for 6 h. Cells were divided into assay and control tubes, each containing 20 μL PE‐CD_4_ antibody, and incubated in the dark for 20 min at room temperature. After washing, cells were treated with fixative solution, permeabilized, centrifuged (1500 rev/min for 5 min), and labeled with 10 μL IL‐17‐PE (assay tube) or 10 μL IgG1‐PE (control tube). Following a 15‐min incubation in the dark, the proportion of Th17 cells among CD4^+^T cells was analyzed by flow cytometry.

Peripheral blood Treg was assessed. An additional 100 μL PBMC suspension was stained with 20 μL FITC‐CD4 and 5 μL PE‐CD25 monoclonal antibodies and incubated in the dark at room temperature for 20 min. Cells were washed, centrifuged, and then permeabilized with 1 mL fixative/permeabilization buffer for 30 min in the dark. Following another wash and centrifugation, cells were resuspended in 100 μL permeabilization buffer, and 200 μL of Foxp3 antibody was added. After a final incubation, cells were analyzed by flow cytometry for the Treg/CD4+ T cell proportion, and the Treg/Th17 ratio was calculated.

### 2.6 Enzyme‐linked immunosorbent assay

Fasting elbow vein blood samples (2 mL) were allowed to clot at room temperature for 30 min, then centrifuged at 3200 rev/min for 8 min to separate the serum, which was stored at −80°C. Serum levels of IL‐17 (QS40180), IL‐6 (QS40049), IL‐10 (QS450711), and TGF‐β1 (QS440053) were measured using ELISA kits (Gersion, Beijing, China) on a Multiskan Mk3 microplate reader (Thermo Fisher Scientific, Waltham, MA, USA).

### 2.7 RNA isolation and reverse transcription quantitative polymerase chain reaction assay

A 3 mL sample of peripheral venous blood was collected at admission or during the physical examination and placed in a dry test tube. After coagulation, serum was obtained by centrifugation (10 cm radius, 3000 rev/min, 4°C, 15 min), and RNA was extracted from the serum. Serum *miR‐135a* expression was measured via qPCR, with *cel‐miR‐39‐3p* as an external reference. Total RNA was extracted using TRIzol, and RNA samples with an absorbance *A*_260_/*A*_280_ ratio of 1.9–2.1 were reverse‐transcribed into complementary DNA (cDNA) using M‐MLV reverse transcriptase (Epicentre, Madison, WI, USA). The RT‐qPCR was then performed on a CFX96 instrument (Bio‐Rad, Richmond, CA, USA) to quantify serum *miR‐135a* expression.

The reaction system set as follows: 12.5 μL SYBR Premix Ex TaqTMII (2×), l.6 μL dNTP, l μL Taq DNA polymerase, 1 μL of upstream and downstream primers (10 μmol/L) each, and reaction buffer up to 20 μL. The PCR cycling conditions were as: pre‐denaturation at 92°C for 20 s, followed by 30 cycles of denaturation at 96°C for 2 s, annealing at 80°C for 6 s, and extension at 85°C for 20 s.

For *cel‐miR‐39‐3p* as the external reference, after RNA extraction, 1 μL of *cel‐miR‐39‐3p* solution (XY‐RDM0000C, BioVendor, Brno, Czech Republic) was added to the samples, reverse‐transcribed into cDNA, and diluted 5‐fold. Then, 2 μL of this cDNA was added to the qPCR reaction system for cycling.^17^ The 2^−ΔCT^ method was used for result analysis. Primer sequences are detailed in Table 1.

**TABLE 1:** Primer sequences.

Gene	Forward (5′–3′)	Reverse (5′–3′)
*miR‐135a*	GCGCCGGTATGGCTTTTTATTCCAT	GTCGTATCCAGTGCAGGGTCCGAGG
*cel‐miR‐39‐3p*	GAGAACACCGGGGAAACAG	TCTTGTGGCCCCTTTGTCG

### 2.8 Statistical analysis

Statistical analyses and visualizations were conducted using SPSS 27.0 (IBM, Armonk, NY, USA) and GraphPad Prism 9.5 (GraphPad Software, San Diego, CA, USA). The Kolmogorov–Smirnov test evaluated data for normal distribution. Normally distributed data were expressed as mean ± standard deviation (SD), and the *t*‐test was used for comparisons between two groups. Non‐normally distributed data were expressed as quartiles [median (minimum, maximum)], and analyzed with the Mann–Whitney *U* test. Categorical data were depicted as counts and percentages, with inter‐group comparisons made using the Chi‐square test. Spearman's correlation coefficient was applied for correlation analysis. The diagnostic value of serum *miR‐135a* for AR was assessed using receiver operating characteristic (ROC) curves, and the cut‐off value along the area under the ROC curve (AUC) were calculated. A significance level of *p* < 0.05 was used for all statistical tests.

## 3 RESULTS

### 3.1 Comparisons of baseline data between the two groups

As shown in Table 2, there were no significant differences in sex, age, BMI, and smoking history between the AR and control groups (all *p* > 0.05). However, the IgE level was significantly higher in the AR group compared to the control group (*p* < 0.05).

**TABLE 2:** Comparison of clinical baseline characteristics.

Item	Control group (*n* = 76)	AR group (*n* = 93)	*p* Value
Sex (male/female)	36/40	45/48	0.895
Age (years)	32.16 ± 9.63	33.85 ± 8.49	0.227
BMI (kg/m^2^)	23.15 ± 3.96	22.75 ± 4.02	0.518
Smoking history (cases, %)	28 (36.84)	40 (43.01)	0.416
IgE (IU/mL)	36.25 ± 9.45	428.56 ± 57.63	<0.001
TNSS score	‐	8 (4,12)	‐
Disease course (years)	‐	5.25 ± 1.58	‐
Symptom onset time	‐		
Intermittent (cases, %)	‐	50 (53.76)	‐
Persistent (cases, %)	‐	43 (46.24)	‐

*Note*: Counting data were expressed as number of cases and percentage, and the Chi‐square test was carried out for inter‐group comparisons; measurement data conforming to normal distribution were presented as mean ± SD, and the *t* test was conducted for comparisons between two groups; non‐normally distributed measurement data were expressed as quartiles, and the Mann–Whitney *U* test was applied for inter‐group comparisons.

Abbreviations: BMI, body mass index; TNSS, total nasal symptom score.

### 3.2 Serum *miR‐135a* level was significantly reduced in AR patients and could diagnose AR

**FIGURE 1:**
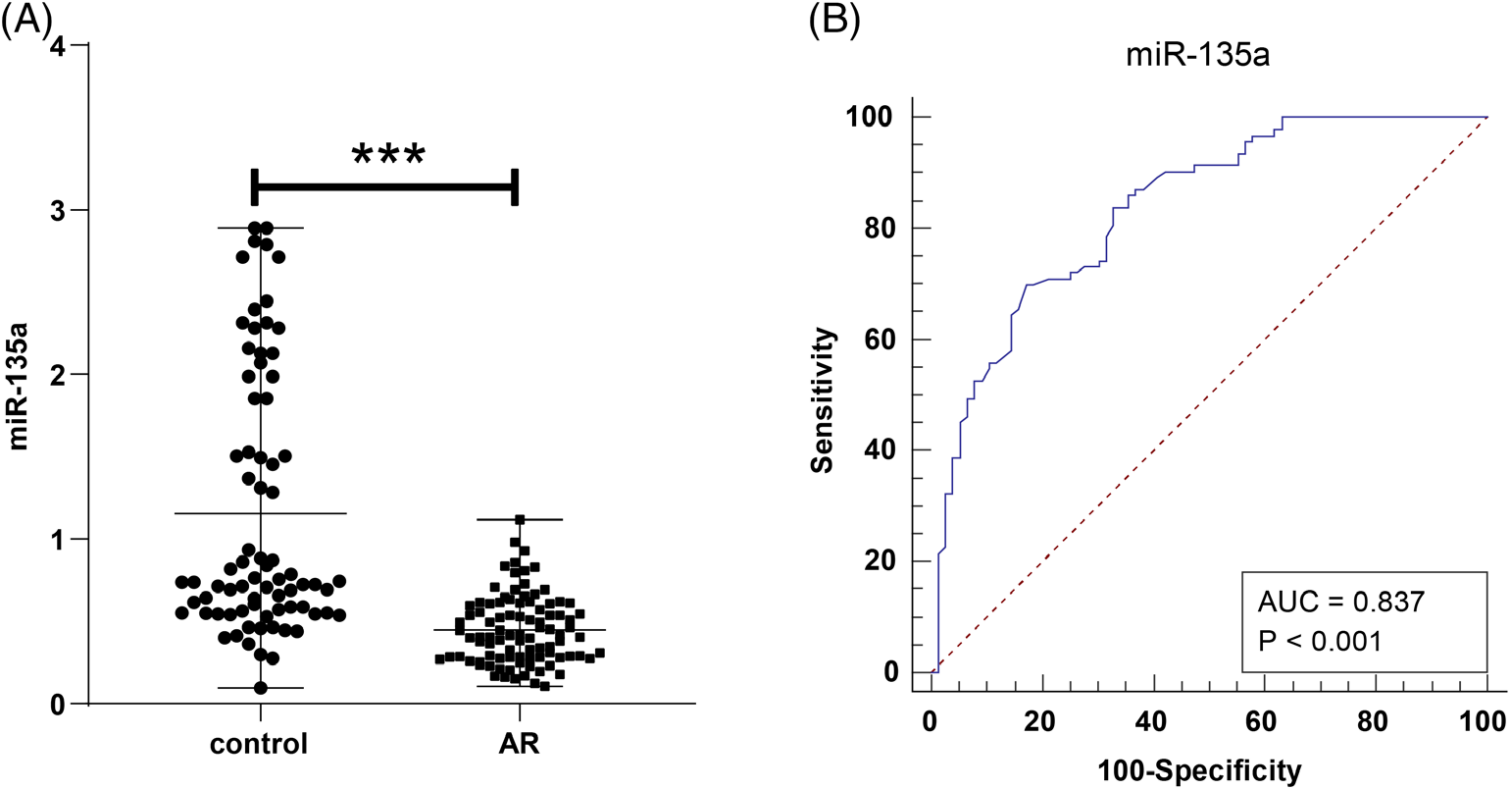
Analysis of serum *miR‐135a* level changes and diagnostic value in AR. (A) Comparison of serum *miR‐135a* levels between the AR and control groups. (B) ROC curve illustrating the diagnostic performance of *miR‐135a* for AR. Serum *miR‐135a* levels were measured using RT‐qPCR, presented as quartiles [median (minimum, maximum)], and inter‐group comparisons were conducted via Mann–Whitney *U* test. ***p < 0.001.

Serum *miR‐135a* levels were markedly lower in the AR group, with a median of 0.415 (0.105–1.117), compared to 0.740 (0.093–2.888) in the control group (*p* < 0.001, Figure 1A). ROC curve analysis indicated that serum *miR‐135a* had diagnostic potential for AR, with an AUC of 0.837 (95% CI 0.772–0.889, a cut‐off value 0.536, sensitivity of 69.89%, and specificity of 82.89%) (Figure 1B).

### 3.3 Treg/Th17 cytokine was imbalanced in AR patients

**FIGURE 2:**
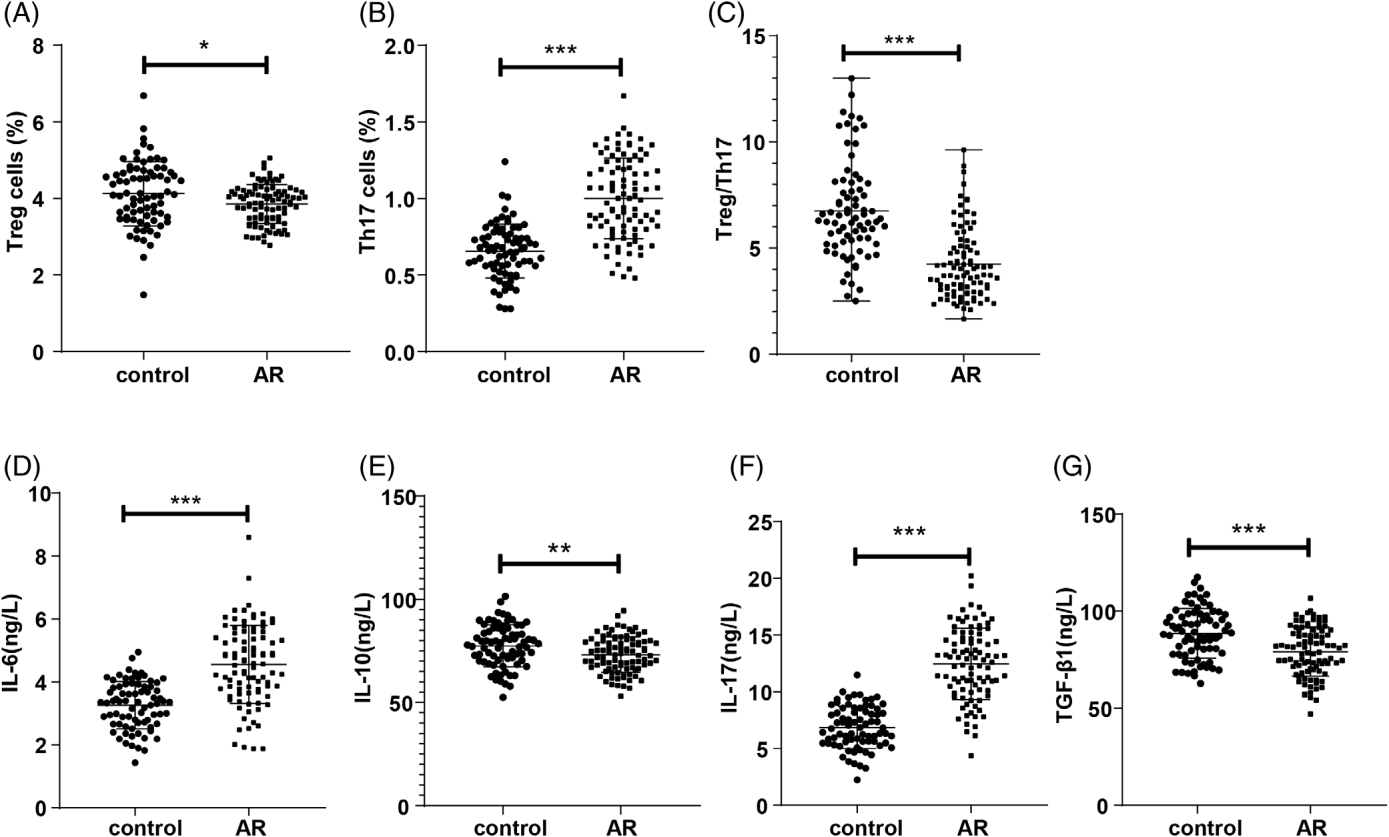
Peripheral blood Th17 and Treg cell levels in AR patients. Data conforming to normal distribution were expressed as mean ± SD, with *t*‐test for inter‐group comparisons. Non‐normally distributed data are shown as median (minimum, maximum), analyzed using the Mann–Whitney *U* test. *p < 0.05, **p < 0.01, ***p < 0.001.

Flow cytometry results revealed an elevated level of peripheral blood Th17 cells, along with reduced Treg cell levels and a lower Treg/Th17 ratio in the AR group compared to the control group (*p* < 0.01, Figure 2A–C). ELISA assessments of Th17/Treg cell‐associated cytokines showed that IL‐17 and IL‐6 levels were significantly increased (*p* < 0.05, Figure 2D,E), while IL‐10 and TGF‐β1 levels were significantly decreased (*p* < 0.01, Figure 2F,G) in the AR group compared to controls.

### 3.4 Correlation of serum *miR‐135a* level with TNSS score and Treg/Th17 balance in AR patients

Patients were divided into high and low *miR‐135a* expression groups (*miR‐135a* ≥ 0.536 and miR‐135a < 0.536, respectively) based on the ROC‐derived cut‐off value. Analysis showed that the TNSS scores were significantly lower in the high miR‐135a expression group compared to the low expression group (*p* < 0.001, Table 3). Furthermore, patients in the high *miR‐135a* expression group exhibited significantly elevated Treg cell levels, a higher Treg/Th17 ratio, and decreased Th17 cell levels relative to the low miR‐135a expression group (all *p* < 0.001, Table 3). Additionally, IL‐10 and TGF‐β1 levels were markedly higher, while IL‐6 and IL‐17 levels were significantly lower in the high *miR‐135a* expression group (all *p* < 0.001, Table 3).

**TABLE 3:** Relationship between serum *miR‐135a* level and Treg/Th17 balance in AR patients.

Item	Low *miR‐135a* expression group (*n* = 65)	High *miR‐135a* expression group (*n* = 28)	*p* Value
TNSS score	10 (5, 12)	5 (4, 10)	<0.001
Th17 (%)	1.11 ± 0.21	0.75 ± 0.19	<0.001
Treg (%)	3.64 ± 0.44	4.34 ± 0.29	<0.001
Treg/Th17 ratio	3.43 ± 0.88	6.13 ± 1.59	<0.001
IL‐17 (ng/L)	13.53 ± 2.66	9.94 ± 2.78	<0.001
IL‐6 (ng/L)	5.04 ± 1.04	3.45 ± 0.93	<0.001
IL‐10 (ng/L)	70.16 ± 7.67	80.10 ± 6.36	<0.001
TGF‐β1 (ng/L)	73.98 ± 10.76	91.14 ± 7.80	<0.001

*Note*: Normally distributed measurement data were expressed as mean ± SD, and comparisons between two groups were conducted using the independent sample *t* test. Non‐normally distributed measurement data were depicted as median value (minimum, maximum), and inter‐group comparisons were conducted using the Mann–Whitney *U* test.

### 3.5 Serum *miR‐135a* level was significantly correlated with TNSS score and Treg/Th17 balance in AR patients

**FIGURE 3:**
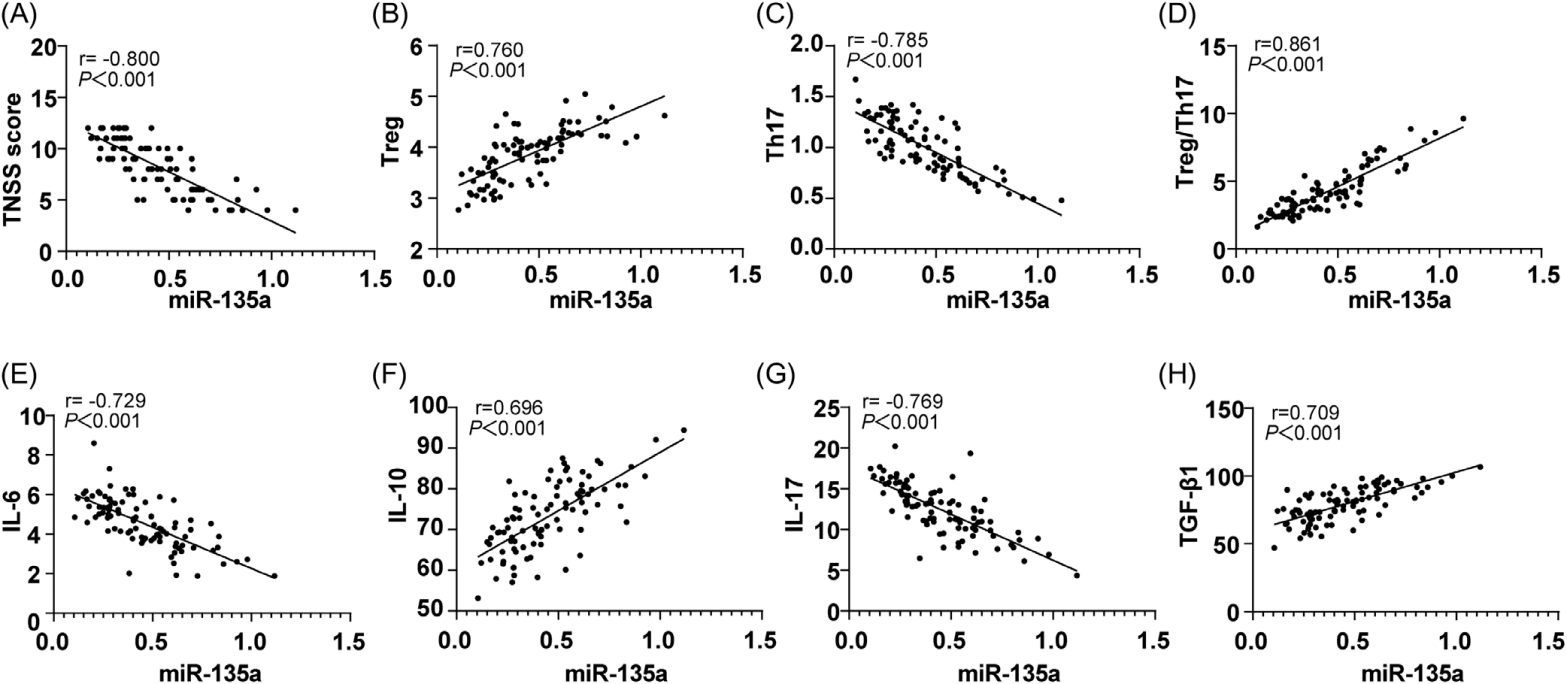
Correlation analysis of serum miR‐135a with TNSS score, Treg, and Th17 in AR patients. Spearman's correlation coefficient was used to examine relationships between serum miR‐135a level, TNSS score, and Treg/Th17‐related cytokines. Serum miR‐135a was measured by RT‐qPCR, Th17, and Treg cell levels were analyzed via flow cytometry, and IL‐17, IL‐6, IL‐10, and TGF‐β1 levels were determined by ELISA.

Spearman's correlation analysis revealed that serum *miR‐135a* levels in AR patients were negatively correlated with TNSS score, IL‐17, IL‐6, and Th17 cell percentage, while positively correlated with IL‐10, TGF‐β1, Treg cell percentage, and the Treg/Th17 ratio (Figure 3, all *p* < 0.001).

## 4 DISCUSSION

Although substantial research has explored the mechanisms underlying AR pathology and treatment, many aspects remain unclear, warranting further investigation.^18^ Recent studies have highlighted the role of epigenetics, such as DNA methylation, miRNA expression, and histone modifications, in AR development.^19^ In this study, we investigated serum *miR‐135a* expression in AR patients and its relationship to Treg/Th17 cell balance.

*miR‐135a*, part of the miR‐135 family (which includes *miR‐135a* and *miR‐135b*), is known for its dysregulation in various cancers.^20^ Emerging evidence also suggests that *miR‐135a* plays a role in regulating inflammation and immune responses.^21^ In asthmatic mouse models, *miR‐135a* overexpression has been shown to alleviate airway inflammation by modulating the JAK/STAT pathway.^22^ In line with these findings, Luo et al. reported a significant reduction in *miR‐135a* levels in the nasal mucosa of AR mice.^15^ Similarly, our study found downregulation of serum *miR‐135a* in AR patients. The ROC analysis further indicated that, at a cut‐off value of 0.536, miR‐135a could serve as a potential biomarker for AR, with a sensitivity of 69.89% and a specificity of 82.89%.

Treg cells are essential for modulating inflammatory responses and maintaining immune tolerance, with Treg and Th17 cells having opposing roles in both function and differentiation.^23^ Imbalance in the Treg/Th17 ratio is closely associated with inflammation.^24^ In AR patients, Th17 cell levels peripheral blood are significantly elevated, while Treg cell levels and the Treg/Th17 ratio are notably reduced.^25^ In line with previous findings, our study confirmed that AR patients exhibited increased Th17 cell levels, along with decreased Treg cell levels and Treg/Th17 ratios in peripheral blood. IL‐10 and TGF‐β are key regulators for Treg cells, while IL‐17 is essential for Th17 cells.^26^, ^27^ Additionally, IL‐6, a pro‐inflammatory cytokine, plays a pivotal role in Treg/Th17 balance regulation.^23^ Previous AR studies in mice have reported elevated IL‐17 and IL‐6 levels, along with decreased IL‐10 levels.^28^ Consistently, we found that peripheral blood IL‐17 and IL‐6 levels were elevated in AR patients, whereas IL‐10 and TGF‐β1 levels were reduced.

The Th17/Treg cell imbalance likely contributes to AR pathogenesis, and modulating these cells may offer a promising therapeutic approach.^29^ miRNAs have emerged as key regulators of Th17/Treg cell balance.^30^ For example, *miR‐181a‐5p* overexpression alleviates allergic symptoms, corrects Treg/Th17 imbalance, and delays asthma onset in AR murine models.^31^ *miR‐146a* overexpression enhances Treg function by increasing TGF‐β and IL‐10 levels in AR patients,^32^ while *miR‐378g* overexpression reduces Th17 cells and IL‐17A levels, while increasing TGF‐β, Treg cell levels, and the Treg/Th17 ratio in AR patients.^33^ Additionally, overexpression of miR‐10b‐5p has been shown to reduce sneezing, rubbing, Th17 cell proportion, IgE, IL‐6, IL‐4, and IL‐17 in AR mice.^34^ Abnormal secretion of immune cells and cytokines, along with Th17, Th2, Treg transcription factors and related cytokines, are implicated in AR progression.^35^ Targeting Th17 cells with an anti‐IL‐17 antibody may enhance CD4+ CD25+ CD127− Treg differentiation, thus alleviating AR symptoms.^36^ Further, miR‐135a‐5p overexpression in lung tissues can significantly reduce IL‐6, IL‐1β, and TNF‐α while increasing IL‐10, thereby modulating inflammatory and oxidative stress responses in acute lung injury.^37^ Previous studies also suggest that miR‐135a may target IL‐17, and downregulation of miR‐135a promotes nasopharyngeal carcinoma development through IL‐17‐mediated cytokine expression.^38^

Our study provided novel insights, demonstrating that serum *miR‐135a* levels in AR patients negatively correlated with IL‐17 and IL‐6 levels and Th17 cell percentages, while showing a positive correlation with IL‐10, TGF‐β1 levels, Treg cell percentages, and Treg/Th17 ratios. The TNSS, a widely used questionnaire to assess AR symptom severity, has shown reductions in scores with symptom alleviation.^39^, ^40^ Intriguingly, we observed a negative correlation between serum miR‐135a levels and TNSS scores in AR patients, suggesting that miR‐135a may influence AR development through these mechanisms.

In summary, our study revealed that serum *miR‐135a* expression is significantly reduced in AR patients and closely associated with Treg/Th17 balance, suggesting its potential as a biomarker for AR diagnosis. However, this study has several limitations. First, it is a single‐center, retrospective study with a relatively small sample size, which may limit the generalizability of the findings. Additionally, the specific mechanisms through which *miR‐135a* influences AR pathogenesis are not yet fully understood. Future research will focus on elucidating the molecular mechanisms underlying *miR‐135a* downregulation in AR. A multi‐center prospective study with an expanded sample size and matched controls will also be conducted to improve diagnostic sensitivity and strengthen the robustness of these findings.

## CONFLICT OF INTEREST STATEMENT

The authors declare no conflict of interest.

## ETHICS STATEMENT

This study was ratified by the Ethics Committee of First Affiliated Hospital of Kunming Medical University and was in line with the Helsinki Declaration.
